# Supplementary material for: Splice-Junction-Based Mapping of Alternative Isoforms in the Human Proteome
Source: Cell Rep. Author manuscript; Available in PMC 2020 Jan 15. (PMC6961840; doi:10.1016/j.celrep.2019.11.026)
Supplement: 2 [file NIHMS1546469-supplement-2.zip › DF1/PXD009737/TestisTrypsin/TestisTrypsin_52_SPTAN1_LPWETAEDIQER.pdf]

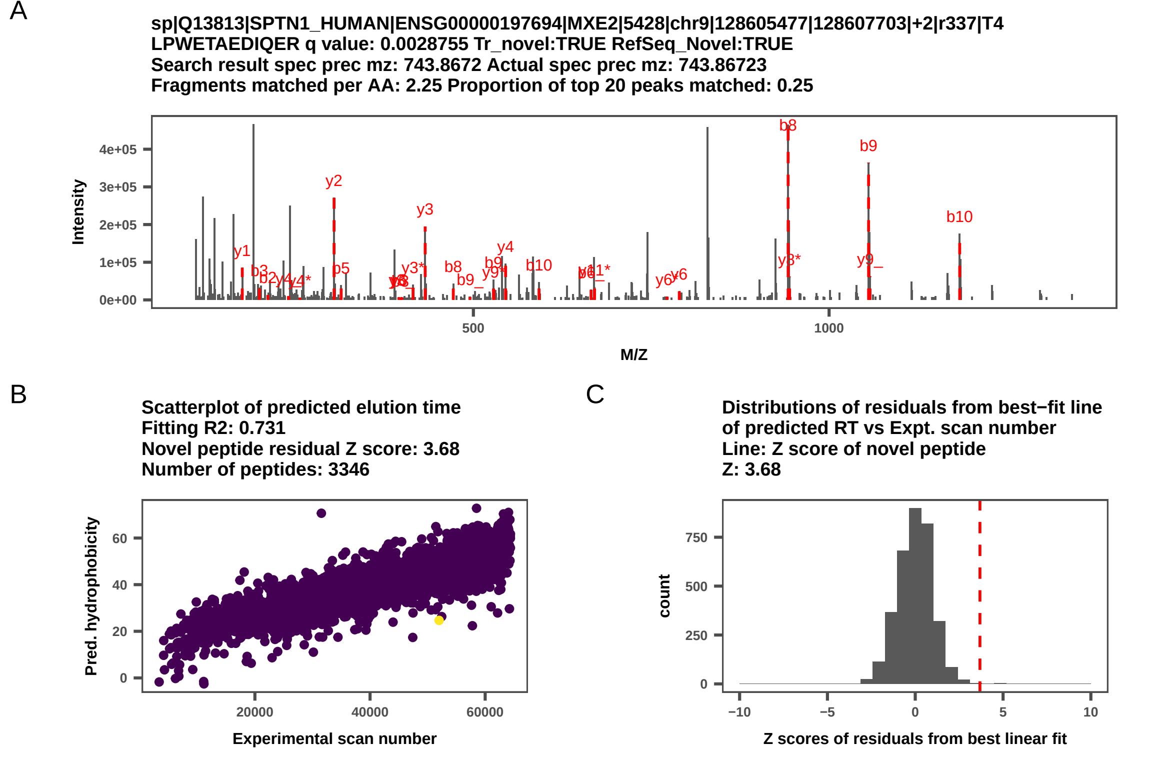
A
sp|Q13813|SPTN1_HUMAN|ENSG00000197694|MXE2|5428|chr9|128605477|128607703|+2|r337|T4
LPWETAEDIQER q value: 0.0028755 Tr_novel:TRUE RefSeq_Novel:TRUE
Search result spec prec mz: 743.8672 Actual spec prec mz: 743.86723
Fragments matched per AA: 2.25 Proportion of top 20 peaks matched: 0.25
Intensity
4e+05
3e+05
2e+05
1e+05
0e+00
M/Z
500
1000
y1
y2
b5
y3
y3*
b8
y4
b9
b10
y6
b8
y8*
b9
b10
B
Scatterplot of predicted elution time
Fitting R2: 0.731
Novel peptide residual Z score: 3.68
Number of peptides: 3346
Pred. hydrophobicity
60
40
20
0
20000
40000
60000
Experimental scan number
C
Distributions of residuals from best−fit line
of predicted RT vs Expt. scan number
Line: Z score of novel peptide
Z: 3.68
count
750
500
250
0
−10
−5
0
5
10
Z scores of residuals from best linear fit
